# A Pilot Study Evaluating the Effectiveness of Platelet-Rich Plasma Therapy for Treating Degenerative Tendinopathies: A Randomized Control Trial with Synchronous Observational Cohort

**DOI:** 10.1371/journal.pone.0147842

**Published:** 2016-02-05

**Authors:** Marni Wesner, Terry Defreitas, Heather Bredy, Louisa Pothier, Ziling Qin, Ashley B. McKillop, Douglas P. Gross

**Affiliations:** 1 Glen Sather Sports Medicine Clinic, University of Alberta, Edmonton, Alberta, Canada; 2 Department of Physical Therapy, University of Alberta, Edmonton, Alberta, Canada; 3 Rehabilitation Research Centre, Faculty of Rehabilitation Medicine, University of Alberta, Edmonton, Alberta, Canada; Cardiff University, UNITED KINGDOM

## Abstract

**Objective:**

This pilot study aimed to inform future research evaluating the effectiveness of Platelet Rich Plasma (PRP) injection for tendinopathy.

**Design:**

Randomized control trial (RCT) and synchronous observational cohort studies. For the RCT, consecutive consenting patients treated at an academic sports medicine clinic were randomly assigned to either a PRP or placebo control group.

**Setting:**

The Glen Sather Sport Medicine Clinic, Edmonton, Canada.

**Patients:**

The RCT included 9 participants with rotator cuff tendinopathy. The cohort study included 178 participants with a variety of tendinopathies.

**Interventions:**

Patients receiving PRP were injected with 4 ml of platelets into the supraspinatus and/or infraspinatus, while patients in the placebo group were injected with 4ml of saline. All participants undertook a 3-month standardized, home-based, daily exercise program.

**Main Outcome Measures:**

Participants in the RCT were re-evaluated 3, and 6 months post-injection. Change scores before and after injection on pain, disability and MRI-documented pathology outcomes were compared. In the cohort study, pain and disability were measured at 1, 2 and 3 months post-injection.

**Results:**

For the RCT, 7 participants received PRP and 2 received placebo injections. Patients receiving PRP reported clinically important improvements in pain (>1.5/10 on VAS), disability (>15 point DASH change), and tendon pathology while those receiving placebo injections did not. In the observational cohort, statistically and clinically significant improvements in pain and disability were observed.

**Conclusion:**

This pilot study provides information for planning future studies of PRP effectiveness. Preliminary results indicate intratendinous, ultrasound-guided PRP injection may lead to improvements in pain, function, and MRI-documented tendon pathology.

**Trial Registration:**

Controlled-Trials.com ISRCTN68341698

## Introduction

Tendinopathy is frequently a painful condition that defines the structural and compositional change in a tendon as a result of cumulative overload leading to a failed healing response.[1, 2] Tendinopathy is associated with pain, disability, depressive symptomology and anxiety.[3–5] The rotator cuff tendons are commonly affected, particularly in individuals over the age of 50.[6–8] Despite mixed and limited evidence, several non-surgical treatment approaches have been clinically used, including non-steroidal anti-inflammatory drugs, eccentric strengthening exercises, corticosteroid injections, Extracorporeal Shock Wave Therapy and platelet rich plasma (PRP) injections.[1]

PRP injections are an emerging therapeutic approach that have been used in a variety of clinical contexts, including dermatology, plastic surgery, sport medicine, dentistry and orthopedic surgery.[9–11] Platelets are cells that contain over 300 bioactive proteins and growth factors that control cell growth and differentiation, synthesis of connective tissue, and revascularization. In tissues that are aging and do not repair or regenerate well, growth factors may help to improve healing of the degenerate tissue by stimulating angiogenesis, epithelialisation, cell differentiation, replication, proliferation and formation of extracellular matrix.

PRP injections have been used as a treatment for tendinopathy and have been shown to positively influence health-relevant outcomes, such as pain and disability.[12–18] Mautner et al. found that following PRP injection, 82% of individuals reported an improvement of greater than 50% in pain symptoms measured with a visual analogue scale (VAS).[15] A recent systematic review of tendinopathy found that pain was significantly reduced up to 1-year following PRP injections.[12] However, methodological quality has varied across the studies, with several limitations commonly observed such as lack of a control group, high attrition rate or small sample size.[12–15, 18]

Research has started to examined the effect of PRP injections on clinical outcomes in individuals with rotator cuff tendinopathy.[6, 15, 16, 18] Of these studies, findings and methodological quality have also varied, making it difficult to conclude if PRP injections are an effective treatment for this population. Rha et al found that individuals who received PRP injections had reduced pain and disability compared to those who received dry needling.[16] A later study by Kesikburun et al. found that PRP injections had no effect on such variables.[6] Prior to conducting a full scale randomized controlled trial to clarify the effectiveness of this treatment for rotator cuff tendinopathy, it is important that pilot studies be conducted to provide background information to help overcome limitations of previous studies. For example, it is unknown why previous PRP studies have had such high attrition rates or small samples, or how best to overcome these problems in future studies.

This pilot project aimed to provide background information to assist in planning large-scale studies aimed at determining the effectiveness of PRP injection on clinical and functional outcomes. Two separate studies were conducted that will be described separately. This included a randomized controlled trial (RCT) aimed at evaluating the effectiveness of a single intratendinous, ultrasound-guided Platelet Rich Plasma (PRP) injection into the degenerate rotator cuff. The second study was an observational cohort study that evaluated patients who received PRP treatment for a variety of tendon disorders.

## Methods and Results

### Study One

#### Design

A pilot prospective, RCT was conducted. This was a parallel design with 1–1 allocation ratio. The study recruitment and follow-up took place between 1 July 2011 and 31 July 2014. The study registration number was ISRCTN68341698 (due to the nature of this study as a pilot study, it was not registered prior to enrolment). The authors confirm that all ongoing and related trials for this intervention are registered.

#### Sample

We aimed to enrol 50 participants, balancing adequate statistical power with the feasibility and expense of collecting Magnetic Resonance Imaging (MRI) data. Thirty consecutive patients with rotator cuff tendinopathy and meeting inclusion criteria treated at the Glen Sather Sport Medicine Clinic (GSSMC) in Alberta, Canada, were assessed for eligibility. Inclusion criteria were: 1) patients between 35 and 60 years of age; 2) minimum of 3-month history of non-traumatic shoulder pain that was unresponsive to previous conservative treatment (including analgesia, subacromial steroid injection and physical therapy); and 3) tendinosis/tendinopathy or partial thickness tears in the supraspinatus and/or infraspinatous tendons confirmed using MRI. All participants had MRI of the affected shoulder prior to referral for consideration of enrollment in the study. No important changes were made to the methods after trial commencement.

Patients were excluded if they had: 1) a history of rotator cuff repair; 2) traumatic injury to the shoulder inducing pathology; 3) MRI findings showing full thickness rotator cuff tear; 4) previous PRP treatment for the rotator cuff; 5) shoulder pain due to a work-related injury/complaint; or 6) diabetes.

Nine eligible patients agreed to participate. These patients were predominantly middle-aged (mean 49 years) males (67%), with moderate levels (4.2/10) of chronic pain (median duration 19 months). Twenty-one patients declined to participate, stating they would prefer to pay for the PRP treatment out of pocket rather than risk receiving placebo treatment. Due largely to this low participation rate, enrolment was stopped.

#### Intervention and Data Collection

**Fig 1:**
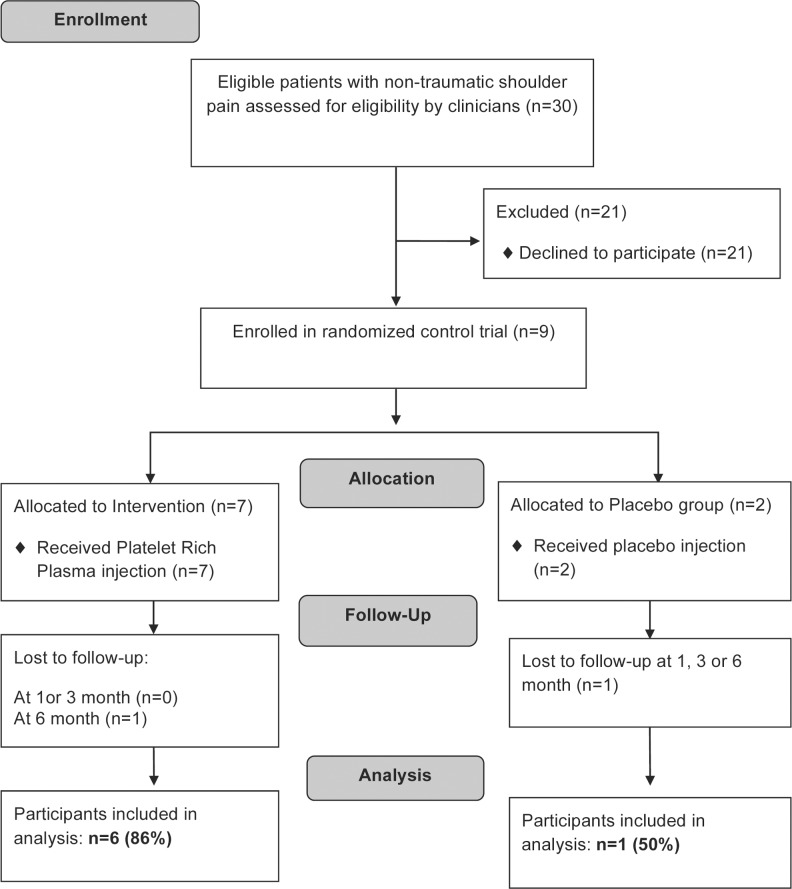
Study Flow Diagram for Platelet-Rich Plasma Injection Pilot Randomized Controlled Trial. Study 1 Flow Diagram.

Patients enrolled in the trial were randomly assigned to either a PRP or placebo control group by pulling group assignment out of a box without replacement (randomization was done by author LP who prepared the injections but was not otherwise involved in patient care). All physicians and the treating physiotherapist were blind to group allocation. At the time of enrolment, all participants had a repeat clinical and ultrasound assessment of the rotator cuff by one of the two physician investigators to correlate ultrasound findings to those demonstrated on MRI. Using the Harvest™ system (Plymouth, Massachusetts) for generating PRP, 4 ml of platelets were fenestrated to the degenerative area of the tendon under ultrasound guidance for each patient in the study group. Patients in the placebo group were injected in the same manner with 4ml of saline. Following the injection, all patients undertook a 3-month standardized, home-based daily exercise program under the supervision of a physical therapist. Patients were clinically re-evaluated at 3, and 6 months post-injection. (See Fig 1 for study flowchart).

#### Measures

Since PRP treatment aims to facilitate tendon healing, reduce pain and increase functional ability, we measured self-report pain intensity and disability using self-report outcome measures as described below, as well as objective clinical evaluations that included assessment of the pre- and post-injection MRI findings. Musculoskeletal radiologists evaluated the MRI results qualitatively and quantitatively where possible using size of the tear in millimeters. MRI was conducted before the injection then repeated 6 months following the study PRP injection. Post-injection findings were compared to pre-injection findings by the assessing radiologist.

Three 10-point VAS measures were used in all patients to measure pain intensity, ability to do daily activities, and physical activity/exercise.[19] These were anchored at 0 with ‘no pain’ and 10 ‘worst imaginable’. These scales were completed before injection and at each follow-up. The minimum clinically important difference for the 10-point VAS was considered to be 1.5/10.[20, 21]

We also measured disability and health-related quality of life using the Disabilities of the Arm Shoulder and Hand (DASH) and Western Ontario Rotator Cuff (WORC) Index questionnaires. These were completed before injection and at each follow-up assessments. The DASH questionnaire is a commonly used, self-report, region-specific outcome instrument developed as a measure of self-rated upper-extremity disability and symptoms.[22] The DASH consists mainly of a 30-item disability/symptom scale, scored from 0 to 100.[23] The Western Ontario Rotator Cuff (WORC) Index questionnaire is a self-report questionnaire consisting of 21 items representing 5 domains to assess disease-specific quality-of-life.[24, 25] There are 6 questions in the physical symptoms domain, 4 in the sports and recreation domain, 4 in the work domain, 4 in the lifestyle domain, and 3 in the emotions domain. All DASH and WORC scores were converted into a standardized score out of 100.[26, 27] Lower DASH scores indicate less disability while higher WORC scores indicate higher life quality. The minimum clinically important difference for the DASH and WORC were considered to be 15/100.[22, 28, 29]

No changes were made to the trial outcomes after the trial commenced.

#### Data analysis

Descriptive statistics were calculated for demographic information and pain and disability scores for all patients. To determine the effectiveness of PRP, we examined overall mean change scores of the VAS, WORC and DASH within subjects and between groups. Data were handled using intention to treat, however statistical testing was not conducted due to limited sample size (originally group comparisons were planned on all of the main outcome measures). Instead, mean change scores were compared to known clinically important differences for the measures. Since only 9 participants enrolled in the trial and the numbers in each group were imbalanced, we considered participants individually and created a comprehensive table of individual results on the various pain and disability measures. All analyses were performed using SPSS version 22.0 (Armonk, New York).

#### Results

Of the 9 participants enrolled in the trial, 7 were randomly assigned to PRP and 2 were assigned to placebo (see Table 1). All participants returned for the 3-month follow-up, however, 1 participant in the placebo group did not return for the 6 month follow-up and did not undertake follow-up MRI. Additionally, 1 participant in the PRP group did not complete the follow-up WORC scale but data were available for all other measures.

**Table 1:** Characteristics and Outcomes of Patients in the Randomized Control Trial.

Patient	Age (years)	Sex	Group	Pain Duration (month)	Follow-up Duration (months)	Pre-Injection VAS* Scores (out of 10)			Mean 6-month VAS Change (positive scores indicate improvement)			Pre-DASH**	Post-DASH	DASH Change	Pre-WORC^#^	Post-WORC	WORC Change
						VAS 1	VAS 2	VAS 3	VAS 1	VAS 2	VAS 3						
1	48	M	PRP	19	6	5	2	7	3	0	1	23	8	-15	52	73	21
2	56	M	PRP	12	6	5	8	7	3	5	5	36	17	-19	52	-	-
3	52	F	Placebo	13	3	4	8	8	-3	-1	-1	67	77	10	25	27	2
4	37	M	PRP	18	6	0	2	10	-1	1	5	26	13	-13	56	82	26
5	48	F	PRP	60	6	6	3	3	5	2	2	34	14	-20	52	84	32
6	56	M	PRP	36	6	0	1	4	-1	1	2	26	8	-18	68	88	20
7	45	M	PRP	360	6	9	8	8	7	6	5	45	9	-36	22	89	67
8	47	M	Placebo	18	6	5	6	6	1	2	2	36	11	-25	39	84	45
9	56	F	PRP	24	6	4	3	6	3	1	2	29	29	0	29	95	66

* Visual Analogue Scales: VAS 1 = Pain intensity, VAS 2 = ability to do daily activities, VAS 3 = physical activity/exercise

** DASH = Disabilities of the Arm, Shoulder, and Hand Questionnaire

^#^ Western Ontario Rotator Cuff Index

Five PRP and 1 placebo participant demonstrated clinically important DASH improvements. Mean change before and after injection on the DASH score was -17.3 (10.7 SD) for the PRP group and -7.5 (24.7 SD) for the placebo group. Six PRP and 1 patient in the placebo group demonstrated clinically important WORC improvements. Mean change on the WORC score was 38.7 (22.0 SD) for the PRP group and 23.5 (30.4 SD) for the placebo group. Mean improvement on the VAS scales was clinically important for the PRP group (2.6, 2.2, 3.0 and 4.1) but not for the placebo group (-1.0, 0.5, 0.5 and 2).

#### MRI Findings

Analysis of the radiologist reporting of the MRI changes identified that 2 participants experienced worsening of the cuff pathology, one participant experienced equivocal change, and 5 of the 8 participants had improvement of the pathoanatomy of the rotator cuff following the treatment (See Table 2). Five out of 7 participants in the PRP group (71%) experienced improved MRI findings after PRP injection, while the participant in the placebo group did not experience improvement. However, the structural changes to the rotator cuff on MRI did not correlate to the pain or functional improvements demonstrated on self-report questionnaires (see Table 1).

**Table 2:** Comparison of MRI Findings Before and After Platelet-Rich Plasma Injections.

	Pre-PRP Findings Reported by Radiologist	6 months Post-PRP Findings Reported by Radiologist	MRI Change Interpretation
Participant 1 (PRP group)	7x4 mm Supraspinatus Tear, 1x3 mm Infraspinatus Tear	3x3 mm Supraspinatus Tear, 1x3 mm Infraspinatus Tear	Better
Participant 2 (PRP group)	Tendinosis of Supraspinatus, Intact Infraspinatus	13 mm Supraspinatus tear, Fraying Infraspinatus	Worse
Participant 3 (Placebo)	Tendinosis of Supraspinatus and Infraspinatus	Lost to follow up	Lost to follow up
Participant 4 (PRP group)	9x2 mm Supraspinatus tear, Intact Infraspinatus	7x2 mm Supraspinatus tear, Intact Infraspinatus	Better
Participant 5 (PRP group)	Calcific Tendonitis Supraspinatus, Normal Infraspinatus	Normal Supraspinatus, Normal Infraspinatus	Better
Participant 6 (PRP group)	2x10 mm Supraspinatus tear, Normal Infraspinatus	Normal Supraspinatus, Minor Tendinosis of Infraspinatus	Better
Participant 7 (PRP group)	Delamination of Supraspinatus, Normal Infraspinatus	Improvement in Delamination	Better
Participant 8 (Placebo)	3 mm Supraspinatus tear, Normal Infraspinatus	11x2 mm Supraspinatus tear, Normal Infraspinatus	Worse
Participant 9 (PRP group)	8 mm Supraspinatus tear, Normal Infraspinatus	8 mm Supraspinatus tear, Questionably More Prominent Tendinosis of Infraspinatus	Equivocal

### Study Two

#### Design

A retrospective observational cohort study was conducted. As mentioned above, over the course of the pilot RCT, we observed that a high proportion (70%) of patients refused to participate in the RCT and preferred to pay out-of-pocket for treatment to ensure they received PRP instead of chance receiving placebo. Therefore, we conducted a retrospective cohort study to observe outcomes in all patients treated at the clinic with PRP over the course of the original trial.

#### Sample

Participants included 178 consecutive patients presenting to the clinic between April 2011 and July 2014 for treatment of tendinopathy-related pain complaints of any body parts. Inclusion criteria included being treated with PRP to any body part and followed-up by physicians at the GSSMC. Patients in this study were predominately males (58%) with a mean age of 44.7 years and moderate pain intensity (5.6/10). Most commonly injected body parts were the Achilles tendon (25%), elbow common extensor tendon (20%), supraspinatus and/or infraspinatus (19%) and patellar tendon (19%).

#### Intervention and Data Collection

**Fig 2:**
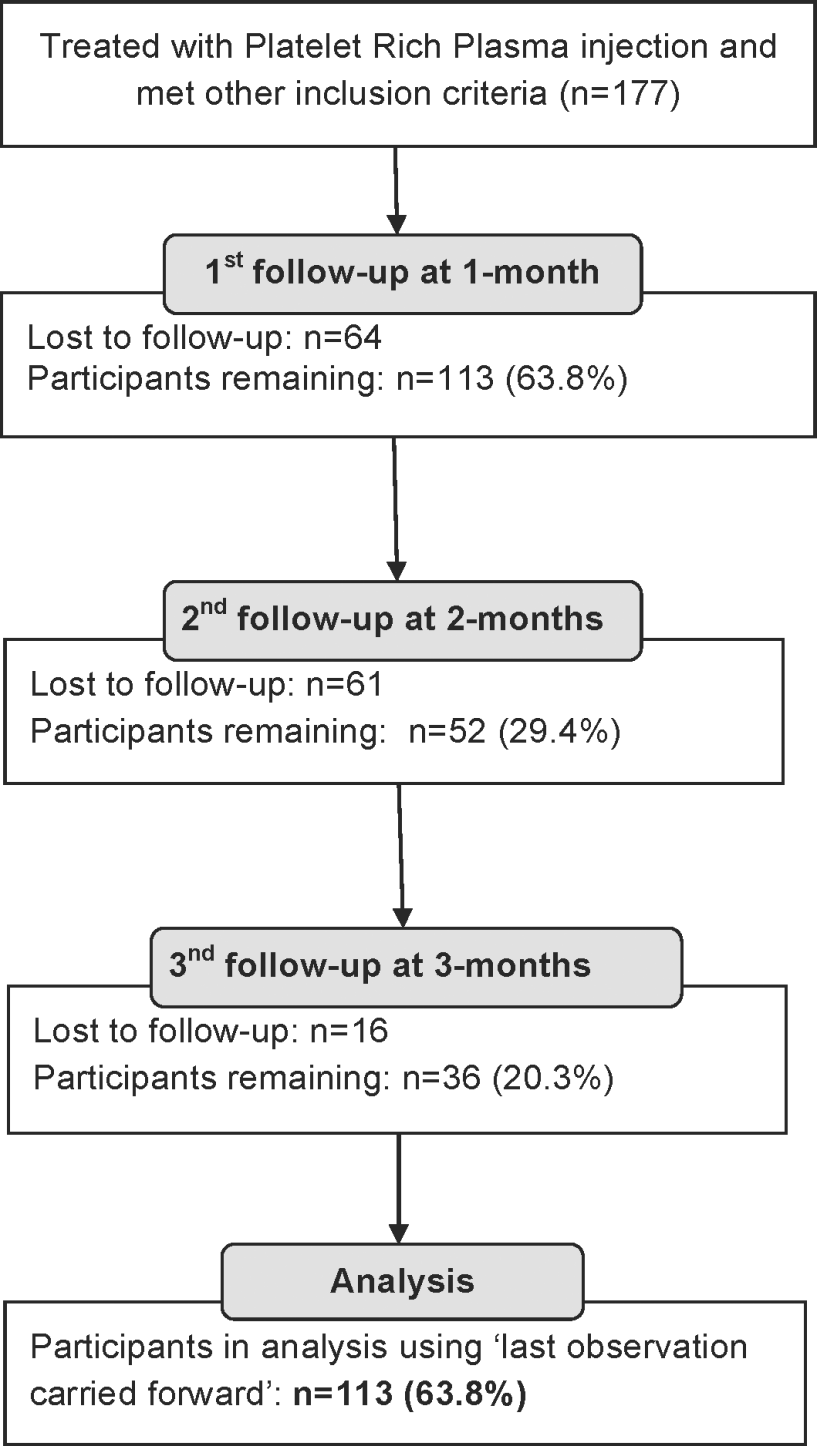
Study Flow Diagram for the Retrospective Observational Study. Study 2 Flow Diagram.

Patients in the observational cohort were treated in a similar manner to the RCT protocol described above. Three to 8ml of PRP were fenestrated to the relevant tendon and patients received a daily, structured home exercise program. These patients were re-evaluated at 1, 2 and 3 months post-injection (See Fig 2 for study flowchart).

#### Measures

Some basic descriptive information was collected including sex, age group, treating doctor, and number of PRP injections received. The number of injections was dependent on the treating doctor, but was typically repeated if response to the initial injection was not of an expected magnitude. In addition to this descriptive information, three 10-point VAS measures were used in all patients to measure pain intensity, ability to do daily activities, and physical activity/exercise.[19] These were anchored at 0 with ‘no pain’ and 10 ‘worst imaginable’. These scales were completed before injection and at each follow-up. The minimum clinically important difference for the 10-point VAS was considered to be 1.5/10.

#### Data analysis

Descriptive statistics were calculated for demographic information and pain and disability scores for all patients. Participants who had incomplete or missing data at baseline were considered missing and were not included in subsequent analysis. Those with partial follow-up were analyzed with listwise deletion as well as with a last observation carried forward strategy.[30] Last observation carried forward provides an unbiased estimate of the magnitude of an effect, if data are missing at random.[30] A comparison was made between those with and without missing data using independent t tests for continuous data and chi-square statistics for nominal data. Pre-injection VAS measurements (pain, function and sports activities) were compared to follow-up VAS scores separately using paired-sample t tests. Additionally, MANOVA was used to determine if there were significant differences in change scores between sex, age groups, number of injections and treating doctor. All analyses were performed using SPSS version 22.0 (Armonk, New York). Alpha level for statistical significance was set at α = 0.05.

#### Results

Of the 178 patients in the observational cohort, 65 participants were excluded due to incomplete data at either baseline or follow-up measures. There were no statistically significant differences between those with and without complete data (See Table 3). The 113 participants (63%) with complete data were predominately male (58%) with a mean age of 44.7 years and had moderate pain intensity (5.6/10). The most commonly injected body parts were the Achilles tendon (25%), elbow (20%), patella tendon (19%) and shoulder (19%). Eighty percent of participants received a single injection while 20% received multiple (2 or 3 injections).

**Table 3:** Characteristics of Patients in the Observational Cohort Study.

Characteristics	Complete Data	Missing Data	
	*Mean (SD) / Number (Percent %)*		*P Value*
	N = 113	N = 65	
Age (years)	44.7 (13.5)	44.1 (12.9)	0.75
Gender (male)	66 (58%)	35 (54%)	0.55
Body Part of Injection			0.96
Achilles	28 (25%)	18 (28%)	
Elbow	23 (20%)	14 (22%)	
Knee	21 (19%)	11 (17%)	
Shoulder	21 (19%)	13 (20%)	
Other	20 (18%)	9 (14%)	
Number of Injections			0.37
1	90 (80%)	55 (85%)	
2	20 (18%)	10 (15%)	
3	3 (3%)	0 (0%)	

Statistically significant and clinically important differences were observed between baseline and follow-up on all VAS measures (See Table 4). Mean improvements on the VAS measures were 2.3 (2.5 SD) for pain intensity VAS (p <0.001), 1.8 (2.5 SD) for limited ability for daily activities VAS (p < 0.001), and 2.7 (2.7 SD) for limited physical activity/exercise VAS (p < 0.001).

**Table 4:** Clinical Outcomes for Patients in the Observational Cohort Study.

**Visual Analogue Scale for Pain Intensity**	**Mean (SD)**	**P Value**
Pre-injection (N = 113)	5.6 (2.4)	
1 month post-injection (N = 113)	3.8 (2.5)	**p < 0.001**
2 month post-injection (N = 52)	3.6 (2.3)	**p < 0.001**
3 month post-injection (N = 36)	3.3 (2.2)	**p < 0.001**
3 month post-injection (LOCF*)	3.3 (2.4)	**p < 0.001**
**Visual Analogue Scale for Daily Activities**		
Pre-injection (N = 113)	4.9 (2.5)	
1 month post-injection (N = 113)	3.5 (2.5)	**p < 0.001**
2 month post-injection (N = 52)	3.4 (2.2)	**p < 0.001**
3 month post-injection (N = 36)	3.1 (2.6)	**p < 0.001**
3 month post-injection (*LOCF*)	3.1 (2.4)	**p < 0.001**
**Visual Analogue Scale for Physical Activity/Exercise**		
Pre-injection (N = 113)	7.2 (1.6)	
1 month post-injection (N = 113)	5.0 (2.6)	**p < 0.001**
2 month post-injection (N = 52)	4.4 (2.1)	**p < 0.001**
3 month post-injection (N = 36)	4.3 (2.7)	**p < 0.001**
3 month post-injection (*LOCF*)	4.5 (2.6)	**p < 0.001**

All post-injection results were statistically compared to pre-injection scores separately.

* LOCF = Results using Last Observation Carried Forward technique.

MANOVA analysis evaluating differences across covariates found that pain intensity VAS and daily activity ability VAS were significantly different between genders (p < 0.05, See Table 5). Females improved more on both pain (2.8 vs. 1.8) and function (2.5 vs. 1.4) compared to males. No other variables showed statistically significant differences among/between other classifications.

**Table 5:** MANOVA Analysis of the Observational Cohort Study Assessing Change Scores Between Pre-Injection and 3-month Post-Injection.

	VAS Pain		VAS Function		VAS Sports Activities	
	*Mean (Standard Deviation) (N = 113)*					
Gender		P Value		P Value		P Value
Male (N = 66)	1.8 (2.5)	0.04*	1.4 (2.5)	0.02*	2.7 (2.7)	0.996
Female (N = 47)	2.8 (2.4)		2.5 (2.5)		2.7 (2.6)	
**Age**						
Under 30 (N = 18)	2.1 (2.8)	0.14	1.1 (2.2)	0.33	2.5 (2.3)	0.31
Between 30 and 60 (N = 84)	2.1 (2.5)		1.9 (2.6)		2.7 (2.7)	
Beyond 60 (N = 11)	3.0 (2.5)		2.9 (2.5)		3.2 (2.7)	
**Body Part**						
Achilles (N = 28)	2.3 (2.7)	0.24	2.4 (2.9)	0.96	2.7 (2.6)	0.45
Elbow (N = 23)	2.3 (2.2)		2.0 (2.3)		2.6 (3.0)	
Knee (N = 21)	1.8 (2.7)		1.1 (2.5)		2.6 (2.5)	
Shoulder (N = 21)	2.6 (2.9)		1.9 (2.7)		2.4 (3.0)	
Other (N = 20)	2.2 (2.2)		1.8 (2.2)		3.3 (2.4)	
**Number of Injections**						
1 (N = 90)	2.2 (2.5)	0.82	1.8 (2.5)	0.73	2.8 (2.6)	0.72
2 (N = 20)	2.6 (2.9)		1.9 (2.5)		2.3 (2.7)	
3 (N = 3)	2.2 (2.5)		3.0 (4.4)		2.5 (3.3)	
**Physician**						
A (N = 40)	2.1 (2.3)	0.36	2.0 (2.4)	0.42	2.7 (2.5)	0.83
B (N = 37)	3.1 (2.5)		2.2 (2.6)		3.3 (2.6)	
C (N = 22)	1.5 (2.8)		1.3 (2.6)		1.8 (2.9)	
Other Doctors (N = 14)	1.6 (2.4)		1.5 (2.7)		2.6 (2.6)	

* Indicates statistically significant differences observed between/among groups.

### Ethical Considerations

The University of Alberta Health Research Ethics Board approved this research (File Numbers: Pro00019481 and Pro00049954, representing separate protocols for the RCT and observational cohort study respectively). There were no modifications to the pilot RCT.

## Discussion

Several observations were made during this pilot study to inform future large-scale RCTs of PRP treatment. Patients considered for this study had very high expectations of PRP treatment, with 70% willing to pay out-of-pocket for the therapy to avoid the possibility of receiving a placebo injection. This presented a serious challenge for recruitment and will likely limit the ability of future investigators to enrol sufficiently large and representative samples of patients for PRP trials. High patient expectations and the placebo effect on clinical outcomes also cannot be discounted.[31] One of the 2 patients in our placebo group demonstrated clinically important VAS scores. This could have been due to a placebo response to the injection, or due to the structured rehabilitation and home exercise program they participated in. Alternative designs that guarantee that patients will receive PRP while still rigourously evaluating effectiveness of the therapy should be considered, such as randomized crossover trials [32] or use of a wait-list control group. Crossover designs also have inherent challenges, such as the duration of acceptable washout period, but they may be more suitable to educated and informed patients who want to experience PRP therapy.

The direction and magnitude of therapeutic effect observed for PRP in this study was quite consistent across patients in the RCT and observational cohort. Clinically important, yet modest improvements were consistently observed. These findings suggest that single intratendinous, ultrasound-guided PRP injection may be effective in improving pain and function in patients with rotator cuff tendinopathy. Interestingly, we observed high rates of MRI-documented improvement in rotator cuff pathology in patients receiving PRP, which was not seen in the patient in the placebo group. However, the improved pathology did not clearly correlate with improved symptoms, a finding that requires further investigation. A moderating variable to be accounted for in future research is gender, as we observed in the cohort study that females demonstrated larger improvements than males. The estimates and standard deviations provided here can be used to estimate sample sizes for future studies.

We found that at one busy academic sports medicine clinic, the number of eligible patients seen meeting our strict inclusion/exclusion criteria was quite low. Over a nearly 3-year enrolment period, we assessed only 30 eligible patients. For the randomized control study, our stringent study criteria focused on patients with confirmed rotator-cuff tendinopathy who had ‘failed’ prior appropriate conservative management. Future studies will either need to have less stringent criteria or have multi-site enrolment to increase sample size.

The current pilot study has several limitations. The sample size for the RCT was too small to conduct inferential statistics. The original study design planned to recruit 50 participants. A large number of patients were screened for potential inclusion in the study, however the vast majority did not meet our strict inclusion criteria and had not attempted or completed what we considered to be appropriate conservative treatment. When conservative treatment was initiated, nearly all patients recovered limiting the number of eligible patients for our study. Nine participants agreed to volunteer in our trial, but 21 other patients meeting the inclusion criteria were not willing to chance receiving a placebo injection and paid out-of-pocket for PRP treatment. Despite not being able to conduct statistical testing with this small sample size, clinically significant results were demonstrated, which was also confirmed in the observational cohort study. Missing data could possibly lead to lack of power and data inconsistency, but we observed little differences across those with complete and incomplete data. Future studies will need to consider providing incentives to improve data collection methods or use different methodological strategies such as a wait-list control group.

### Conclusion

This pilot study provides important information for planning future studies of PRP effectiveness. High patient expectations for PRP are likely to influence enrollment and alternative designs such as crossover studies should be considered. Preliminary results from this study indicate a single, intratendinous, ultrasound-guided PRP injection lead to improvements in pain, function, and MRI-documented tendon pathology in patients with rotator cuff tendinopathies.

## Supporting Information

S1 File CONSORT Checklist. (DOCX)

S2 File Full Study Protocol for Randomized Control Trial (Study 1). (PDF)

S3 File Full Study Protocol for Observational Cohort Study (Study 2). (PDF)
