# Supplementary material for: A Pilot Study Evaluating the Effectiveness of Platelet-Rich Plasma Therapy for Treating Degenerative Tendinopathies: A Randomized Control Trial with Synchronous Observational Cohort
Source: PLoS One. 2016 Feb 5;11(2):e0147842. doi: 10.1371/journal.pone.0147842 (PMC4743976; doi:10.1371/journal.pone.0147842)
Supplement: S2 File — (PDF) [file pone.0147842.s002.pdf]

Date:

ID: Pro00019481

Status: Assigned To REB Meeting

{hidden}

{hidden} {hidden} {hidden} {hidden}

Print Close

View: 1.1 Study Identification

ID:Pro00019481

Status: Assigned To REB Meeting

1.1 Study Identification

*All questions preceded by a red asterisk * are required fields. Other fields may be required by the REB in order to evaluate your application.*

Please answer all presented questions that will reasonably help to describe your study or proposed research.

1.0 * **Short Study Title** *(restricted to 250 characters)*:
The effect of platelet rich plasma injection to the age-related degenerate rotator cuff

2.0 * **Long Study Title** *(can be exactly the same as short title)*:
The effect of platelet rich plasma injection to the age-related degenerate rotator cuff

3.0 * **Select the appropriate Research Ethics Board:**
HREB Biomedical

4.0 * **Which office requires notification of ethics approval to release funds or finalize the study contract?** *(It is the PI's responsibility to provide ethics approval notification to any office other than the ones listed below)*
Not applicable

5.0 * **Name of Principal Investigator** *(at the University of Alberta, Covenant Health, or Alberta Health Services)*:
Marni Wesner

6.0 **Investigator's Supervisor** *(Required for graduate students and trainees NOT applying to the Health Research Ethics Board (HREB). The HREBs do not accept graduate students or trainees as Principal Investigators in an ethics application. Please enter your supervisor as the PI and yourself as a co-investigator in your application for HREB.*
Supervisor is required if the PI is a student researcher.

7.0 * **Type of research/study:**
Faculty/Staff Research

8.0 **Study Coordinators/Assistants** *(will have access to and can edit this application and will receive all notifications for this study)*:

Name	Employer
	There are no items to display

9.0 **Co-Investigators (*Authorized List*):** The following people can act as co-authors to this application: they will have access to, and can edit, this ethics application online. Co-investigators do not receive HERO notifications about the progress of the applications unless they are added to the study email list.

Name	Employer
	There are no items to display

Study Team *(co-investigators, supervising team, other study team members who do not require access to this application or to receive notifications)***:**

10.0

Last Name	First Name	Organization	Role	Phone	Email
Bredy	Heather	Glen Sather Sport Medicine Clinic	co-investigator	780-492-4752	heather.bredy@ualberta.ca
Defreitas	Terry	Glen Sather Sport Medicine Clinic	co-investigator	780-492-4752	reif_defreitas@shaw.ca

ID: Pro00019481 View: 1.2 Additional Approval

Status: Assigned To REB Meeting

1.2 Additional Approval

1.0 * **Departmental Review:**
MH Family Medicine

2.0 **Internal Review:**

ID: Pro00019481 View: 1.3 Funding Information

Status: Assigned To REB Meeting

1.3 Study Funding Information

1.0 * **Type of Funding:**

Grant (external to the institution)

Your answer here will deteremine the next form you will see.

If OTHER, provide details:

Funding Source

2.0 **2.1 Select all sources of funding from the list below:**
There are no items to display

2.2 If not available in the list above, write the Sponsor/Agency name(s) in full (you may add multiple funding sources):
View workman's compensaton board

3.0 **Location of funding source** *(required if study is funded)***:**
Canada

RSO University-Managed Funding

4.0 **4.1 If your funds are managed by the Research Service Office (RSO), select the project ID and title from the lists below to facilitate release of your study funds.** *(Not available yet)*

4.2 If not available above, provide all identifying information about the study funding:

Project ID	Project Title	Speed Code	Other Information
	There are no items to display		

ID: Pro00019481 View: 1.4 Conflict of Interest
Status: Assigned To REB Meeting

1.4 Conflict of Interest

1.0 *** Are any of the investigators or their immediate family receiving any personal remuneration (including investigator payments and recruitment incentives but excluding trainee remuneration or graduate student stipends) from the funding of this study that is not accounted for in the study budget?**

(hidden) (hidden) Yes (hidden) (hidden) **No**

If YES, explain:

2.0 *** Do any of investigators or their immediate family have any proprietary interests in the product under study or the outcome of the research including patents, trademarks, copyrights, and licensing agreements?**

(hidden) (hidden) Yes (hidden) (hidden) **No**

3.0 **Is there any compensation for this study that is affected by the study outcome?**

(hidden) (hidden) Yes (hidden) (hidden) **No**

4.0 **Do any of the investigators or their immediate family have equity interest in the sponsoring company? (This does not include Mutual Funds)**

(hidden) (hidden) Yes (hidden) (hidden) **No**

5.0 **Do any of the investigators or their immediate family receive payments of other sorts, from this sponsor (i.e. grants, compensation in the form of equipment or supplies, retainers for ongoing consultation and honoraria)?**

(hidden) (hidden) Yes (hidden) (hidden) **No**

6.0 **Are any of the investigators or their immediate family, members of the sponsor�s Board of Directors, Scientific Advisory Panel or comparable body?**

(hidden) (hidden) Yes (hidden) (hidden) **No**

7.0 **Do you have any other relationship, financial or non-financial, that, if not disclosed, could be construed as a conflict of interest?**

(hidden) (hidden) Yes (hidden) (hidden) **No**

If YES, explain:

Important

If you answered YES to any of the questions above, you may be contacted by the REB for more information or asked to submit a Conflict of Interest Declaration.

ID: Pro00019481 View: 1.5 Study Locations and Sites
Status: Assigned To REB Meeting

1.5 Study Locations and Sites

1.0 * **Specify research locations: Enter all locations where the research will be conducted under this Research Ethics Approval** *(eg. university site, hospital, community centre, school, classroom, participant�s home, in the field, clinician�s private office, internet website, etc. - provide details)*:
Glen Sather Sport Medicine Clinic, University of Alberta

2.0 * **Please check if your study will utilize or access facilities, programmes, resources, staff, students, specimens, patients or their records, at any of the sites affiliated with the following** *(select all that apply):*
Not applicable

Details must be provided if Alberta Health Services and/or Covenant Health and/or Capital Care selected:

3.0 **If the study involves researchers in other institution(s), will ethics approval be sought from other institutions/organizations** *(eg. another university, Alberta Cancer Board, school district board, etc)*?
Not Applicable

If YES, provide a list:

Name
There are no items to display

ID: Pro00019481 View: 2.1 Study Objectives and Design
Status: Assigned To REB Meeting

2.1 Study Objectives and Design

1.0 **Proposed Start Date:**
01/06/2011

2.0 **Proposed start date for working with human participation** *(can be the same as item 1.0)*:
01/06/2011

3.0 **Proposed end date for working with human participation:**
01/06/2013

4.0 * **Provide an abstract or lay summary of your proposed research** *(restricted to approx. 300 words)*:

106 patients aged 35-65 who have shoulder pain complaints referable to age-related rotator cuff degeneration with MRI demonstrated tendinopathy but no cuff tears will be invited to participate in a randomized controlled trial investigating the efficacy of a single, ultra-sound guided platelet rich plasma injection into the rotator cuff (compared to saline) to resolve pain complaints and improve function. 53 patients in the study and control groups will be enrolled. All participants will undergo a 3 month structured and supervised period of phyiscal therapy, and VAS, the WOMC and DASH scales will be measured at time 0, 1,3,6,and 12 months post-PRP injection to determine the sustainability of results. MRI will be repeated 6 months after the PRP to determine objective resolution or change to the area of tendinosis of the cuff.

5.0 * **Provide a description of your proposed research** *(study objectives, background, scope, methods, procedures, etc) (restricted to approx. 1,000 words)*:

The natural, age-related degenration of the rotator cuff is an exceedingly common problem that causes complaints of shoulder pain in the 35-65 year age group. In the absence of full-thickness rotator cuff tears (RCT) management of the aging cuff can be difficult to effect complete resolution of pain and improve function. Platelet-rich plasma (PRP) injection is an emerging therapeutic option in sport medicine to manage soft tissue injury. PRP is a safe, effective procedure that uses the patients own blood to generate a concentrated source of platelets. Platelets have over 300 active growth factors and moderate and improve the healing response in soft tissue injury. In the degenerate tendon, PRP stimulates tenocyte proliferation, angiogenesis and diminshes fibrosis of the aging tendon. PRP has been used very successfully to treat degenerate Achilles and patellar tendons, the common extensor group at the elbow. PRP has also been used intra-operatively after a repair of a rotator cuff tear to improve the healing response and speed recovery. To

our knowledge, it has not been used with ultrasound guidance into the area of tendinosis identified on magnetic resonance imaging (MRI) or ultrasound (US) imaging. 106 patients (53 control, 53 study group) between ages 35 and 65 and complaining of a painful shoulder with clinical findings of shoulder impingement syndrome and and MRI demonstraing tendinosis but no partial or full thickness tears to the rotator cuff will be recruited, and randomly assigned to the control or study group. The study group will have 30 ml of blood drawn from the anticubital vein. Using the Biomet system, 3 ml of PRP will be generated. Using US guidance, the PRP will be injected into the area of tendinosis of the rotator cuff. The control group will have 3ml of saline injected to the tendinopathic area of the cuff. Study participants will be blinded to the randomization. All patients will have the shoulder immobalized in a sling for 2 days after the injection, followed by participation in a structured physical therapy program for 3 months. Preceeding the injections, a visual analogue scale to assess level of pain, as well as the Disabilty of the Arm, Shoulder and Hand (DASH) and the Western Ontario Rotator Cuff (WORC) scales will be completed to objectively assess pain and function. The VAS, DASH and WORC will be repeated at 3, 6 and 12 months after the PRP injection to assess sustainabilty of results. The MRI will be repeated at 6 months after PRP injection to determine objective alterations to the tendinopathic area of the cuff on imaging. Given the publised success with the use of PRP in other areas of tendinosis in the body, it is anticipated that PRP in the intact but degenerate rotator cuff will effect healing and improvement in the appearane of the cuff on MRI and improvement in pain and functional use of the shoulder.

6.0 **Describe procedures, treatment, or activities that are above or in addition to standard practices in this study area** *(eg. extra medical or health-related procedures, curriculum enhancements, extra follow-up, etc)***:**

none

7.0 **If this research proposal has received independent scientific or methodological review, provide information** *(eg. names of committees or individuals involved in the review, whether review is in process or completed, etc)***:**

n/a

8.0 **If this application is related to or builds upon a previously approved application at the University of Alberta, please provide the study title and ethics file/approval number or any other reference if available:**

n/a

ID: Pro00019481 View: 3.1 Risk Assessment

Status: Assigned To REB Meeting

3.1 Risk Assessment

1.0 * **After reviewing the Minimal Risk Criteria provided in User Help, provide your assessment of the risk classification for this study:**

Minimal Risk

2.0 * **In a scale of 0 to 10 where 0 = No Likelihood, 5 = Moderate Likelihood and 10 = Extreme Likelihood, put a numerical rating in response to each of the following:**

Rate	Description of Potential Risks and Discomforts
0	Psychological or emotional manipulations will cause participants to feel demeaned, embarrassed, worried or upset
0	Participants will feel fatigued or stressed
0	Questions will be upsetting to the respondents
1	Participants will be harmed in any way
0	There will be cultural or social risk � for example, possible loss of status, privacy, and/or reputation
1	There will be physical risk or physiological manipulations, including injury, infection, and possible intervention side-effects or complications
0	The risks will be greater than those encountered by the participants in everyday life

3.0 * **Provide details of short- and long-term risks and discomforts:**

Short term risk includes infection to the shoudler from the PRP injection, but this is not any greater than the similar risk for steroid injection to the area, which is a commonly accepted treatment option. Short term (i.e 1-2 days) there is pain associated with PRP injection but this is tollerable with the use of tylenol. There are no long term risks with this study.

4.0 * **Describe how you will manage and minimize risks and discomforts, as well as mitigate harm:**

Patients are fully informed regarding the expected period of discomfort following the procedure. Tylenol can be used if necessary. Pain settles in 1-2 days after PRP. Patients will be fully informed and provided written direction regarding possible signs of infection to the joint and will be instructed to followup up immediately with the study physicians or

through emergeny (if the medical clinic is not open).

5.0 *** If your study has the potential to identify individuals that are upset, distressed, or disturbed, or individuals warranting medical attention, describe the arrangements made to try to assist these individuals. Explain if no arrangements have been made:**
all participants will be advised to return to the Glen Sather Sport Medicine Clinic from Mon-Fri 7 am-7 pm if they have any concerns regarding the procedure and follwup. Outside this time, they will be directed to the emergency room.

ID: Pro00019481 View: 3.2 Benefits Analysis
Status: Assigned To REB Meeting

3.2 Benefits Analysis

1.0 **Describe any benefits of the proposed research to the participants:**
improvement in pain and function in activity of daily life, sleep and work

2.0 *** Describe the scientific and/or scholarly benefits of the proposed research:**
This study will document another treatment option and efficacy for degenerative rotator cuff, outline treatment protocol and rehabilitation protocol

3.0 **Describe any benefits of the proposed research to society:**
Shoulder pain from the degnerative cuff is exceedingly common... if we can improve this a great many people will have a more comfortable shoulder and better use/function of the joint.

4.0 **Benefits/Risks Analysis - describe the relationship of benefits to risk of participation in the research:**
The expected benefit outweighs the risk. PRP has been very effective in resolving tendinosis and pain in other areas of the body and has been used intraoperatively to improve soft tissue healing. The only risk is for infection from the injection but the improvement to pain and function is expected to be significant or total. What is not know is the durabilty of the response.

ID: Pro00019481 View: 4.1 Participant Information
Status: Assigned To REB Meeting

4.1 Participant Information

1.0 **Describe and justify the inclusion criteria for participants (eg. age range, health status, gender, etc):**

35 is the age at which the rotator cuff begins to age/degenerate, and by age 50, as many as 50% of persons can be demonstrated to have frank rotator cuff tears simply based on the aging process. Even more have pain complaints with no identifiable pathology on imaging. Particpants aged 35-60 will be included in this study. Participants can not have had prior surgery for thier complaints, and will have tried traditional treatment options and still complain of pain. The n=106 (53 control, 53 study) was chosen to reflect the number needed to determine power in the results to demonstrate a 10% improvement in outcome, which is a value that is significant to the Workman's Compensaton Board with respect to claim closure and return to work. Male and female participants will be enrolled, and if the soulder complaints are bilateral, only one shoulder will be used for the study (i.e. injected with PRP) and the other may be used as control (i.e. injected with saline.)

2.0 **Describe and justify the exclusion criteria for participants:**

Excluded from the study are patients age less than 35 or greater than 60, those with partial or full thickness rotator cuff tears, work-injured patients and those with prior treatment with PRP in the cuff or surger to the shoulder.

3.0 **Are there any direct recruitment activities for this study?**
( ) (hidden) ( ) (hidden) Yes (•) (hidden) (•) (hidden) **No**
Participants

4.0 **Total number of participants you expect to enroll** *(including controls, if applicable)*:
106

Of these how many are controls, if applicable *(Possible answer: Half, Random, Unknown, or an estimate in numbers, etc)*.

53

If this is a multi-site study, how many participants *(including controls, if applicable)* **do you anticipate will be enrolled in the entire study?**

106

Justification for sample size:

5.0 This study was submitted to the WCB Alberta for funding, since the results are highly appropriate to the working population complaining of shoulder pain. A 10% reduction in claim time and return to work is significant to the WCB. This study sample size was powered to create a minimum diffence in outcome between study and control groups of 10%.

If possible, provide expected start and end date of the recruitment/enrollment period:

6.0 **Expected Start Date: 01/06/2011**

Expected End Date: 01/06/2012

**ID:**	Pro00019481	View: 4.2 Recruit Potential Participants
**Status:**	Assigned To REB Meeting	

4.2 Recruit Potential Participants

Recruitment

1.1 Will potential participants be recruited through pre-existing relationships with researchers *(eg. employees, students, or patients of research team, acquaintances, own children or family members, etc)***?**

1.0 ○ (hidden) ○ (hidden) Yes ◉ (hidden) ◉ (hidden) **No**

1.2 If YES, identify the relationship between the researchers and participants that could compromise the freedom to decline (eg. professor-student). How will you ensure that there is no undue pressure on the potential participants to agree to the study?

Outline any other means by which participants could be identified *(eg. response to advertising such as flyers, posters, ads in newspapers, websites, email, listservs; pre-existing records or existing registries; physician or community organization referrals; longitudinal study, etc)***:**

2.0 Participants will be recruited from all of the sport medicine and orthopedic surgeon practices at the Glen Sather Sport Medicine Clinc. Posters advertising the research project will be posted in the GSSMC exam rooms, and all sport and orthopedic medicine physicians/surgeons will be advised of the study inclusion criteria. Anyone meeting the inclusion criteria will be advised of eligibiltiy for the study but will voluntarily enroll in the project.

**ID:**	Pro00019481	View: 4.3 Recruitment Contact Methods
**Status:**	Assigned To REB Meeting	

4.3 Recruitment Contact Methods

How will initial contact be made? Select all that apply:

1.0 Potential participants will contact researchers

Contact will be made through an intermediary

If contact will be made through an intermediary *(including snowball sampling)***, select one of the following:**

2.0 Intermediary provides information to potential participants who then contact the researchers

If contact will be made through an intermediary, explain why the intermediary is appropriate and describe what steps will be taken to ensure participation is voluntary:

3.0 There are potentially 16 physicians/surgeons who will have patients referred to them that may be appropriate for the study. The treating physician/surgeon will determine if the patient is fit for the study and advise the patient of the pro/cons and risk/benefit of the study. If the patient wishes to participate in the study, the patient will be assessed by one of the two studyphysicians and included in the research project.

Provide the locations where participants will be recruited, *(i.e. educational institutions, facilities in Alberta Health*

4.0 *Services or Covenant Health, etc)***:**

Glen Sather Sport Medicine Clinic

ID: Pro00019481 View: 4.4 Informed Consent Determination

Status: Assigned To REB Meeting

4.4 Informed Consent Determination

1.0 * **Describe who will provide informed consent for this study:**
All participants will be competent to give informed consent

2.0 **How is consent to be indicated and documented?**
Signed consent form

3.0 **What assistance will be provided to participants, or those consenting on their behalf, who have special needs** *(eg non-English speakers, visually impaired, etc)***:**
Only participants who understand English will be enrolled, visually impaired participants will have consent explictly described and verbal consent will be witnessed.

4.0 **If at any time a participant wishes to withdraw or not participate in certain aspects of the research, describe the procedures and the last point at which it can be done:**
The participants may withdraw from the study at any time. Incomplete data collected prior to withdrawal will be included in the final analysis. This is a prospective study investigating the sustainability of the outcomes 2 years after the treatment. Loss of long term follow up is anticipated but all data will be included for analysis.

5.0 **Describe the circumstances and limitations of data withdrawal from the study, including the last point at which it can be done:**
Data withdrawal will affect the long term follow up with respect to sustainabilty of the outcome and may alter the statistical analysis.

6.0 **Will this study involve an entire group where non-participants are present?**
○ (hidden) ○ (hidden) Yes ◉ (hidden) ◉ (hidden) **No**

7.0 **Describe the incentives and/or reimbursements, if any, to participants and provide justification:**
No reimbursement will be offered to participants. PRP has significant beneficial outcomes when used for chronic tendinopathy at other areas of degenerate tendon causing pain. The incentive to participate is a potentially improved outcome and resolution of pain that will be more sustained than traditional conservative/non-surgical treatment.

ID: Pro00019481 View: 4.8 Study Population Categories

Status: Assigned To REB Meeting

4.8 Study Population Categories

1.0 * **This study is designed to TARGET or specifically include the following** *(does not apply to co-incidental or random inclusion)***. Select all that apply:**
Women
Men

ID: Pro00019481 View: 5.1 Research Methods and Procedures

Status: Assigned To REB Meeting

5.1 Research Methods and Procedures

1.0 * **This study will involve the following** *(select all that apply)*
The list only includes categories that trigger additional page(s) for an online application. For any other methods or procedures, please indicate and describe in your research proposal in the Study Summary, or provide in an attachment:
Health and Biological Specimen Collection

2.0 **Does this study involve a Clinical trial** *(includes any research study that prospectively assigns human participants or groups of humans to one or more health-related intervention(s) to evaluate the effects on health outcomes; does not include randomized controlled trials � RCT � outside of clinical settings)?*

(hidden) (hidden) **Yes** (hidden) (hidden) No

3.0 **For registered clinical trial(s), provide registry and registration number, if available:**

Internet-based research

4.1 Will you be doing any internet-based research that involves interaction with participants?

(hidden) (hidden) Yes (hidden) (hidden) **No**

4.0 **4.2 If YES, will these interactions occur in private spaces (eg. members only chat rooms, social networking sites, email discussions, etc)?**

(hidden) (hidden) Yes (hidden) (hidden) No

4.3 Will these interactions occur in public space(s) where you will post questions initiating and/or maintaining interaction with participants?

(hidden) (hidden) Yes (hidden) (hidden) No

5.0 **If you are using any tests in this study diagnostically, indicate the member(s) of the study team who will administer the measures/instruments:**

	Test Name	Test Administrator	Organization	Administrator's Qualification
View	shoulder ultrasound	Dr. Defreitas and/or Wesner	Glen Sather Sport Medicine	CME and personal study for the clinical use of ultrasound assessment of soft tissue/shoulder
View	MRI	radiologist at MIC College Plaza		radiologist

6.0 **If any test results could be interpreted diagnostically, how will these be reported back to the participants?**
The MRI results will be interpreted by the radiologists at MIC College Plaza. The US will be used only as a clinical tool to guide the PRP injection into the degenerate area of the cuff by either Dr. Defreitas or Dr. Wesner.

5.2 Clinical Trial

Protocol

1.0 **1.1 Protocol Number, if applicable** *(if you don�t know what this is, you don�t have one and that�s okay)*:

1.2 Protocol Date:

Is this an investigator-initiated clinical trial?

2.0 * **Is this study authored, initiated and conducted by a member of the University of Alberta, Alberta Health Services and/or Covenant Health faculty or staff?**

(hidden) (hidden) **Yes** (hidden) (hidden) No

* **Is this study authored or funded by a commercial sponsor including, but not limited to, a pharmaceutical company or clinical research organization?**

(hidden) (hidden) Yes (hidden) (hidden) **No**

3.0 ***Does the study involve any of the following?**

Answer	Description
○ (hidden) ○ (hidden) Yes ◉ (hidden) ◉ (hidden) **No**	A drug, device, biologics, vaccine or natural health product not marketed in Canada?
○ (hidden) ○ (hidden) Yes ◉ (hidden) ◉ (hidden) **No**	A comparative bioavailability trial?
○ (hidden) ○ (hidden) Yes ◉ (hidden) ◉ (hidden) **No**	Use of a marketed drug, device, biologics, vaccine, or natural health product outside the parameters of its officially �approved use� by Health Canada?

If you have answered yes to any of the questions above, a Health Canada Clinical Trial Application (CTA) may be required. Please contact NACTRC at 407-3809 for assistance. The Investigator is responsible to coordinate with NACTRC for all Health Canada clinical trials.

4.0 **Trial Phase:**
There are no items to display

5.0 **If applicable, describe the provisions made to break the code of a double-blind study in an emergency situation, and indicate who has the code:**
This study will be blinded to the participant and the study physical therapist, but the study physicians will not be blinded.

6.0 **If applicable, provide justification for using placebo or no-treatment arm:**
A control group of 53 participants will have the degenerative cuff injected with normal saline. The study group will have PRP injected to the degenerative cuff. The control group is needed to compare outcome/results of PRP to the improvement and sustainable results of the study treatment.

7.0 **If applicable, describe the clinical criteria for withdrawing an individual subject from the study due to safety or toxicity concerns:**
Any participant that sustains an infection in the shoulder as a result of either the saline or PRP injection will be withdrawn from the study.

ID: Pro00019481 View: 5.4 Data Safety and Monitoring for Clinical Trials
Status: Assigned To REB Meeting

5.4 Data Safety and Monitoring for Clinical Trials

1.0 * **Check one that most accurately reflects the plan for data safety and monitoring for this study:**
The study will be monitored only by the study investigators.

2.0 * **Describe data monitoring procedures while research is going on. Include details of planned interim analysis, Data Safety Monitoring Board, or other monitoring systems:**
All participants will complete the Disability of Arm Shoulder and Hand (DASH) and the Western Ontario Rotator Cuff (WORC) questionnaires and a visual analogue pain scale (VAS) before the saline/PRP injection, and again at the 3,6,12,18 and 24 months post treatment.

3.0 * **Summarize any pre-specified criteria for stopping or changing the study protocol due to safety concerns:**
n/a

ID: Pro00019481 View: 5.11 Health and Biological Specimen Collection
Status: Assigned To REB Meeting

5.11 Health and Biological Specimen Collection

1.0 * **Indicate health or biological specimen(s) that will be collected** *(for example, body tissues or fluids, be specific)***:**
30-60 ml of blood will be drawn from the participants arm and spun in the Biomet centrifuge to produce 3-6 ml of PRP which will be injected back into the degenerative cuff. There is no risk for transfusion reaction or blood born illness as it

is the patients own blood that is used to generate the PRP.
* **This study will involve the following** *(select all that apply)*:

2.0 Collection of sample for immediate use

If OTHER, provide details:

Explain how the specimen will be collected:
3.0 phlebotomy from anticubital vein into sterile syringe and transfered to commerciall available Biomet system for generation of platelet-rich plasma.
Explain how the specimen will be stored, and for how long:
4.0 There is no storage of specimen, rather is injected immediately upon production/separation of the PRP. the platelet-poor plasma and the red cells are immediately discarded in biohazzard waste recepticals.
Specify all intended uses of collected specimen:
5.0 The platelet-rich plasma is injected into the degenerate cuff. The platelet-poor plasma and red cells are disposed of according to appropriate biohazardous waste disposal.

ID: Pro00019481 View: 5.13 Biohazard Safety
Status: Assigned To REB Meeting

5.13 Biohazard Safety

AMENDMENT OR RENEWAL: If this application is for the amendment or renewal of a pre-existing clinical study, have new biohazards and/or manipulations been added to the research that were not identified in the original study protocol?

1.0 * **NOTE:** If this application is for a new study or a pre-existing non-clinical study, please select "Not Applicable".
Not Applicable

If you selected **NO**, this amendment or renewal is exempt from requiring further review by the EHS Biosafety Division and the *original biohazard approval remains valid*. You do not need to respond to any of the questions below.
Will your research involve the use of one or more of the following? Provide a response for each item.

2.0

				Answer						Description
○	(hidden)	○	(hidden)	Yes	◉	(hidden)	◉	(hidden)	**No**	Risk group 2, 3 or 4 viruses, bacteria, fungi, parasites or eukaryotic cell lines
○	(hidden)	○	(hidden)	Yes	◉	(hidden)	◉	(hidden)	**No**	Environmental specimens suspected to contain risk group 2, 3 or 4 microbes
○	(hidden)	○	(hidden)	Yes	◉	(hidden)	◉	(hidden)	**No**	Large-scale single volume culture in excess of 10 litres for any microbe or eukaryotic cell line
○	(hidden)	○	(hidden)	Yes	◉	(hidden)	◉	(hidden)	**No**	Microbial toxins
◉	(hidden)	◉	(hidden)	**Yes**	○	(hidden)	○	(hidden)	No	Human clinical specimens, including blood or other body fluids, or primary culture of human cells
○	(hidden)	○	(hidden)	Yes	◉	(hidden)	◉	(hidden)	**No**	Xenotransplant studies involving vertebrate donors

(hidden) (hidden) Yes	(hidden) (hidden) **No**	and/or recipients
		Genetic therapy studies involving vertebrate donors and/or recipients
(hidden) (hidden) Yes	(hidden) (hidden) **No**	Genetic manipulation involving virulence genes from risk group 2, 3 or 4 microbes, mammalian oncogenes, mammalian cytokine or interleukin genes, or microcide resistance genes

If you have any questions or concerns regarding the biohazard approval process, please contact:

Dr. Daniel Dragon
Biosafety Officer
Environmental Health and Safety
780-492-3142
EHS_RSR@ehs.ualberta.ca
Campus Mail: MS Code 287

In a cover letter, be sure to include the Principal Investigator�s name and department, the project title, and the name of the grant, if applicable

6.1 Data Collection

1.0 * **Will the study team know the participants� identity at any stage of the study?**
(hidden) (hidden) **Yes** (hidden) (hidden) No

2.0 **Primary/raw data collected will be** *(check all that apply)***:**
Confidential
Coded

3.0 **If identifying information will be removed at some point, when and how will this be done?**
identifying information will be removed at analysis of the data. Since these will be patinets in active treatment at the Glen Sather Sport Medicine Clinic, they will be known to thier treating physician during the course of care. When treatment is complete and the data is entered into the research study, the identifying information will be removed and the data for analysis will be coded, and only reveal the patient's age and sex for inclusion in the demographic results of the study.

4.0 **If this study involves secondary use of data, list all sources:**

5.0 **In research where total anonymity and confidentiality is sought but cannot be guaranteed** *(eg. where participants talk in a group)* **how will confidentiality be achieved?**

6.2 Data Identifiers

1.0 * **Personal Identifiers:** will you be collecting any of the following *(check all that apply)*:

Full Name
Address
Full Postal Code
Telephone Number
Full Date of Birth
Year of Birth
Age at time of data collection

If OTHER, please describe:
the identifying data is the demographical information on the medical chart contained at the Glen Sather Sport Medicine Clinic

2.0 **Will you be collecting any of the following** *(check all that apply)*:

Health Care Number

If OTHER, please describe:

3.0 **If you are collecting any of the above, provide a comprehensive rationale to explain why it is necessary to collect this information:**
Patients will be recruited from the Glen Sather Sport Medicine Clinic(GSSMC) and will be treated through the GSSMC. Name, date of birth, health care number, address and phone numbers are collected as part of the GSSMC demongraphical information on the patients charts. This will be accessible to the study physicians as the information collected for the study will be part of the active treatment received from the GSSMC.

4.0 **Specify information that will be RETAINED once data collection is complete, and explain why retention is necessary. Include the retention of master lists that link participant identifiers with de-identified data:**
The only data retained will be the clinical chart notes and demongraphical data. This will be retained for the specified period of time necessary to retain medical charts.

5.0 **If applicable, describe your plans to link the data in this study with data belonging to another organization:**
n/a

6.3 Data Confidentiality and Privacy

1.0 * **How will confidentiality of the data be maintained? Explain the steps you propose to maintain data confidentiality and privacy.** *(For example, study documents must be kept in a locked filing cabinet and computer files encrypted, etc.)*
The data is retained as a part of the electronic medical records at the GSSMC. The Healthquest electronic health record is password protected and creates an electronic trail of every access to the chart. The EMR is retained on the GSSMC computers/servers in the locked clinic, in the locked building of the University of Alberta Physical Education building. The DASH, WORC and VAS paper files will be scanned into the EMR of the GSSMC and the original paper questionnaires will be kept in a locked file cabinet for the requisite period of data retention.

2.0 **What privacy education/training do members of the team have prior to their access to data?**
The GSSMC physicians and physical therapist have used this Heathquest system for 6 years and training is not necessary. The research assistant will be advised of the necessity of confientiality of the data and will sign the GSSMC's student oath of confidentialty to protect the data.

3.0 **If you involve colleagues, assistants, transcribers, interpreters and/or other personnel to carryout specific research tasks in your study, how will you ensure that they properly understand and adhere to the University of Alberta**

standards of data privacy and confidentiality?
The research assistant/statician will be advised of the necessity for confidentiality an will sign the GSSMC Oath of Confidentialty.
Data Access

*** 4.1 Will the researcher make raw data that identify individuals available to persons or agencies outside of the research team?**
(hidden) (hidden) **Yes** (hidden) (hidden) **No**

4.0 **4.2 If YES, describe in detail what identifiable information will be released, to whom, why they need access, and what safeguards will be used to protect the identity of subjects and the privacy of their data.**

4.3 Provide details if identifiable data will be leaving the institution, province, or country *(eg. member of research team is located in another institution or country, etc.)*
n/a

6.4 Data Storage, Retention, and Disposal

Where will the research data be stored? Specify the physical location and how it will be secured to protect confidentiality.
1.0 Data will be stored at the Glen Sather Sport Medicine Clinic in the the confidential electronic medical record. Paper files will be stored in a locked file cabinet in the GSSMC.
Describe what will happen to the data once the study is completed. Indicate your plans for the destruction of the identifiers at the earliest opportunity consistent with the conduct of the research and/or clinical needs:
2.0 The paper files (DASH, WORC, VAS) will be kept for the requisite period of time for data retention. The electronic medical record will be kept for the necessary period of retention as dictated by the College of Physicians and Surgeons of Alberta.
You must keep your data for a minimum of 5 years according to GFC Policy 96.2. How will you provide for data security during this time?
3.0 The paper files of the DASH, WORC and VAS questionnaires will be kept in a locked file cabinet as well as scanned into the electronic medical record at the Glen Sather Sport Medicine Clinic.

7.1 Documentation

Add documents in this section according to the headers. Use Item 12.0 "Other Documents" for any material not specifically mentioned below.

Sample templates are available in the HERO Home Page in the **Forms and Templates**, or by clicking HERE.

Important: Please do not use .docx files as attachments. It is recommended you convert these files first to .doc (standard Word document files) before attaching.

1.0 **Recruitment Materials:**

Document Name	Version	Date	Description

	Document Name	Version	Date	Description
	participant letter CUFF PRP.doc.docx | History	0.02	18/02/2011 6:31 PM	
	recruitment poster | History	0.01	20/12/2010 6:22 PM	

2.0 **Letter of Initial Contact:**

Document Name	Version	Date	Description
participant letter CUFF PRP.docx | History	0.02	18/02/2011 6:32 PM	

3.0 **Informed Consent / Information Document(s):**

3.1 What is the reading level of the Informed Consent Form(s):

Grade 8

3.2 Informed Consent Form(s)/Information Document(s):

Document Name	Version	Date	Description
participant letter CUFF PRP.docx | History	0.02	18/02/2011 6:32 PM	

4.0 **Assent Forms:**

Document Name	Version	Date	Description
	There are no items to display		

5.0 **Questionnaires, Cover Letters, Surveys, Tests, Interview Scripts, etc.:**

Document Name	Version	Date	Description
	There are no items to display		

6.0 **Protocol:**

Document Name	Version	Date	Description
	There are no items to display		

7.0 **Investigator Brochures/Product Monographs** *(Clinical Applications only)***:**

Document Name	Version	Date	Description
	There are no items to display		

8.0 **Health Canada No Objection Letter** *(NOL)***:**

Document Name	Version	Date	Description
	There are no items to display		

9.0 **Confidentiality Agreement:**

Document Name	Version	Date	Description
	There are no items to display		

10.0 **Conflict of Interest:**

Document Name	Version	Date	Description
	There are no items to display		

11.0 **Other Documents:**

For example, Study Budget, Course Outline, or other documents not mentioned above

Document Name	Version	Date	Description
WCB research grant application.docx | History	0.01	13/02/2011 2:40 PM	

ID: Pro00019481 View: SF - Final Page

Status: Assigned To REB Meeting

Final Page

You have completed your ethics application! Please select "Exit" to go to your study workspace.

This action will NOT SUBMIT the application for review.

Only the Study Investigator can submit an application to the REB by selecting the "SUBMIT STUDY" button in My Activities for this Study ID:Pro00019481.

You may track the ongoing status of this application via the study workspace.

Please contact the REB Administrator with any questions or concerns.

(hidden)
