# Supplementary material for: A Pilot Study Evaluating the Effectiveness of Platelet-Rich Plasma Therapy for Treating Degenerative Tendinopathies: A Randomized Control Trial with Synchronous Observational Cohort
Source: PLoS One. 2016 Feb 5;11(2):e0147842. doi: 10.1371/journal.pone.0147842 (PMC4743976; doi:10.1371/journal.pone.0147842)
Supplement: S3 File — (PDF) [file pone.0147842.s003.pdf]

Date: Tuesday, December 8, 2015 10:53:35 AM

Print Close

VIEW000072

1.1 Study Identification

*All questions marked by a red asterisk * are required fields. However, because the mandatory fields have been kept to a minimum, answering only the required fields may not be sufficient for the REB to review your application.*

Please answer all relevant questions that will reasonably help to describe your study or proposed research.

1.0 * **Short Study Title** *(restricted to 250 characters)*:
Ultrasound Guided Platelet Rich Plasma Injection for Tendon Injuries: A Retrospective File Review

2.0 * **Complete Study Title** *(can be exactly the same as short title)*:

Ultrasound Guided Platelet Rich Plasma Injection for Tendon Injuries: A Retrospective File Review

3.0 * **Select the appropriate Research Ethics Board (***Detailed descriptions are available by clicking the HELP link in the upper right hand corner of your screen***):**
HREB Panel B

4.01 * **Is the proposed research:**
Unfunded

5.0 * **Name of Principal Investigator** *(at the University of Alberta, Covenant Health, or Alberta Health Services)*:
Marni Wesner

6.0 **Investigator's Supervisor** *(required for applications from undergraduate students, graduate students, post-doctoral fellows and medical residents to Boards 1, 2, 3. HREB does not accept applications from student PIs)*

7.0 * **Type of research/study:**
Faculty/Staff Research

8.01 **Study Coordinators or Research Assistants:** People listed here can edit this application and will receive all HERO notifications for the study:

Name	Employer
There are no items to display	

9.01 **Co-Investigators:** People listed here can edit this application but do not receive HERO notifications unless they are added to the study email list:

Name	Employer	Employer.ID

Douglas Gross RM Physical Therapy 3403001

10.01 **Study Team** *(Co-investigators, supervising team, other study team members):* People listed here cannot edit this application and do not receive HERO notifications:

Last Name	First Name	Organization	Role/Area of Responsibility	Phone	Email
Pothier	Louisa	GSSMC	Director		louisa.pothier@ualberta.ca
Bredy	Heather	Department of Physical Therapy	Clinician Scientist		hbredy@ualberta.ca
DeFreitas	Terry	GSSMC	Clinician Scientist		drterrytkd@hotmail.com

1.2 Additional Approval

1.0 * **Departmental Review:**

MH Family Medicine

RM Physical Therapy

2.0 **Internal Review:**

1.5 Conflict of Interest

1.0 * **Are any of the investigators or their immediate family receiving any personal remuneration (including investigator payments and recruitment incentives but excluding trainee remuneration or graduate student stipends) from the funding of this study that is not accounted for in the study budget?**

○ Yes ● **No**

If YES, explain:

2.0 * **Do any of investigators or their immediate family have any proprietary interests in the product under study or the outcome of the research including patents, trademarks, copyrights, and licensing agreements?**

Yes **No**

3.0 * **Is there any compensation for this study that is affected by the study outcome?**
Yes **No**

4.0 * **Do any of the investigators or their immediate family have equity interest in the sponsoring company? (This does not include Mutual Funds)**
Yes **No**

5.0 * **Do any of the investigators or their immediate family receive payments of other sorts, from this sponsor (i.e. grants, compensation in the form of equipment or supplies, retainers for ongoing consultation and honoraria)?**
Yes **No**

6.0 * **Are any of the investigators or their immediate family, members of the sponsor's Board of Directors, Scientific Advisory Panel or comparable body?**
Yes **No**

7.0 * **Do you have any other relationship, financial or non-financial, that, if not disclosed, could be construed as a conflict of interest?**
Yes **No**

If YES, explain:

Important
If you answered YES to any of the questions above, you may be contacted by the REB for more information or asked to submit a Conflict of Interest Declaration.

1.6 Research Locations and Other Approval

1.0 * **List the locations of the proposed research, including recruitment activities. Provide name of institution or organization, town, or province as applicable**
Glen Sather Sports Medicine Clinic

2.0 * **Indicate if the study will use or access facilities, programmes, resources, staff, students, specimens, patients or their records, at any of the sites affiliated with the following** *(select all that apply):*

Not applicable

List all facilities or institutions as applicable:

3.0

Multi-Institution Review

* **3.1 Has this study already received approval from another REB?**
○ **Yes** ◉ **No**

4.0

Does this study involve pandemic or similar emergency health research?
○ Yes ◉ **No**

If YES, are you the lead investigator for this pandemic study?
○ Yes ◉ **No**

5.0 **If this application is closely linked to research previously approved by one of the University of Alberta REBs or has already received ethics approval from an external ethics review board***(s)***, provide the HERO study number, REB name or other identifying information. Attach any external REB application and approval letter in Section 7.1.11 – Other Documents.**
This project is linked to Project: PRO00019481_REN3 Efficacy of Ultrasound Guided Platelet Rich Plasma Injection for Rotator Cuff Degeneration

2.1 Study Objectives and Design

1.0 **Date that you expect to start working with human participants:**
7/7/2014

2.0 **Date that you expect to finish working with human participants, in other words, you will no longer be in contact with the research participants, including data verification and reporting back to the group or community:**
12/31/2014

3.0 * **Provide a lay summary of your proposed research suitable for the general public** *(restricted to 300 words)*. **If the PI is not affiliated with the University of Alberta, Alberta Health Services or Covenant Health, please include institutional affiliation.**
An emerging therapeutic procedure for both acute and chronic soft tissue injuries is the use of platelet rich plasma (PRP) injections. For some, the aging process in tendons causes significant problems of pain and limited function, for which the traditional conservative treatment measures do not offer sufficient relief. PRP aims to facilitate healing, reducing pain and increasing function. Previous literature has documented favourable results using PRP for tendinopathy affecting the patellar and Achilles tendons. However, a review of the literature suggests that ultrasound guided intratendinous use of

platelet rich plasma has not been sufficiently studied to date. This study considers the effect of PRP injections on various tendinopathies when applied using ultrasound guided methodology. We will perform a retrospective file review of patients seen at the Glen Sather Sports Medicine Clinic between October 13, 2012 to present. This includes 243 patients with injuries of the shoulder, knee, and ankle. We will statistically examine reported pain and functional ability before and after PRP injection to the affected site.

4.0 * **Provide a description of your research proposal including study objectives, background, scope, methods, procedures, etc)** *(restricted to 1000 words)*. **Footnotes and references are not required and best not included here. Research methods questions in Section 5 will prompt additional questions and information.**

OBJECTIVE
To evaluate the effectiveness of PRP injections on various tendinopathies for reducing pain and increasing function.

BACKGROUND AND SCOPE
An emerging therapeutic procedure for both acute and chronic soft tissue injuries is the use of platelet rich plasma (PRP) injections. For some, the aging process in tendons causes significant problems of pain and limited function, for which the traditional conservative treatment measures do not offer sufficient relief. PRP aims to facilitate healing, reducing pain and increasing function. Previous literature has documented favourable results using PRP for tendinopathy affecting the patellar and Achilles tendons. However, a review of the literature suggests that ultrasound guided intratendinous use of platelet rich plasma has not been sufficiently studied to date. This study considers the effect of PRP injections on various tendinopathies.

METHODS
Design: We will perform a retrospective file review of patients seen at the Glen Sather Sports Medicine Clinic (GSSMC) between October 13, 2012 to present. This includes 243 patients with injuries of the shoulder, knee, and ankle.
Subjects: All patients were referred to the GSSMC for treatment with PRP. They were examined by sports medicine physicians and if found suitable to the treatment, underwent PRP injection by a trained physician. They then underwent a standardized rehabilitation program as part of the therapeutic regime.
Measures: All patients were evaluated pre-injection with standardized physical examination of strength and range of motion, pain Visual Analogue Scales and reported functional abilities. They were followed-up 6 weeks later with similar measures. All these measures will be extracted from clinic files. We will also extract any relevant qualitative information about functional abilities reported to physicians and recorded on the patient chart.
Analyses: Descriptive statistics will be calculated. Differences between pre- and post-injection strength, range of motion, pain and functional ability will be analyses using t tests.

IMPLICATIONS
This study will provide important clinical information about the effectiveness of PRP injections for painful shoulder, knee and ankle injuries. Results will serve as background information to inform a broader trial of PRP treatment at the clinic.

5.0 **Describe procedures, treatment, or activities that are above or in addition to standard practices in this study area** *(eg. extra medical or health-related procedures, curriculum enhancements, extra follow-up, etc)*:
None - this is a chart review study of patients who have already been seen at the clinic.

6.0 **If the proposed research is above minimal risk and is not funded via a competitive peer review grant or industry-sponsored clinical trial, the REB will require evidence of scientific review. Provide information about the review process and its results if appropriate.**
Not applicable.

7.0 **For clinical research only, describe any sub-studies associated with this application.**
None planned.

3.1 Risk Assessment

1.0 * **Provide your assessment of the risks that may be associated with this research:**
Minimal Risk - research in which the probability and magnitude of possible harms implied by participation is no greater than those encountered by participants in those aspects of their everyday life that relate to the research (TCPS2)

2.0 * **Select all that might apply:**

Description of Potential Physical Risks and Discomforts

No	Participants might feel physical fatigue, e.g. sleep deprivation
No	Participants might feel physical stress, e.g. cardiovascular stress tests
No	Participants might sustain injury, infection, and intervention side-effects or complications
No	The physical risks will be greater than those encountered by the participants in everyday life

Potential Psychological, Emotional, Social and Other Risks and Discomforts

No	Participants might feel psychologically or emotionally stressed, demeaned, embarrassed, worried, anxious, scared or distressed, e.g. description of painful or traumatic events
No	Participants might feel psychological or mental fatigue, e.g intense concentration required
Possibly	Participants might experience cultural or social risk, e.g. loss of privacy or status or damage to reputation
No	Participants might be exposed to economic or legal risk, for instance non-anonymized workplace surveys
No	The risks will be greater than those encountered by the participants in everyday life

3.0 * **Provide details of the risks and discomforts associated with the research, for instance, health cognitive or emotional factors, socio-economic status or physiological or health conditions:**

Since this is a retrospective medical chart review, the only risk is potential social risk from a breach of confidentiality. However, all data will be anonymously handled and the investigative team will avoid presenting identifying information in publications and presentations.

4.0 * **Describe how you will manage and minimize risks and discomforts, as well as mitigate harm:**
All data will be anonymized and the investigative team will avoid presenting and identifying information in publications and presentations.

5.0 * **If your study has the potential to identify individuals that are upset, distressed, or disturbed, or individuals warranting medical attention, describe the arrangements made to try to assist these individuals. Explain if no arrangements have been made:**
Not applicable - retrospective file review.

3.2 Benefits Analysis

1.0 * **Describe any potential benefits of the proposed research to the participants. If there are no benefits, state this explicitly:**
No benefits to patients.

2.0 * **Describe the scientific and/or scholarly benefits of the proposed research:**
As PRP is an emerging therapeutic procedure that has been minimally studied to date, this project will provide important information about its effectiveness in a clinical setting. Results will also inform the planning of a broader RCT of PRP effectiveness.

3.0 **Benefits/Risks Analysis: Describe the relationship of benefits to risk of participation in the research:**
As the associated risks are minimal, benefits outweigh the risk.

4.1 Participant Information

1.0 * **Who are you studying? Describe the population that will be included in this study.**
243 patients with shoulder, knee, elbow or ankle pain who have been treated at the GSSCM with PRP injection.

2.0 * **Describe the inclusion criteria for participants (e.g. age range, health status, gender, etc.). Justify the inclusion criteria (e.g. safety, uniformity, research methodology, statistical requirement, etc)**

All adult patients treated at the clinic with PRP between October 13, 2012 to present will be included. No other restrictions will be placed on the sample.

3.0 **Describe and justify the exclusion criteria for participants:**

Children <18 years of age.

4.0

* **Will you be interacting with human subjects, will there be direct contact with human participants, for this study?**

◯ Yes ◉ **No**

Note: No means no direct contact with participants, chart reviews, secondary data, interaction, etc.

If NO, is this project a chart review or is a chart review part of this research project?

◉ **Yes** ◯ No

5.0

Participants

How many participants do you hope to recruit *(including controls, if applicable)*
243
Of these how many are controls, if applicable *(Possible answer: Half, Random, Unknown, or an estimate in numbers, etc).*
0
If this is a multi-site study, for instance a clinical trial, how many participants *(including controls, if applicable)* **are expected to be enrolled by all investigators at all sites in the entire study?**

6.0 **Justification for sample size:**
We will extract information on all patients treated with PRP. 243 should provide ample sample size for our planned statistical analysis.

7.0 **Does the research specifically target aboriginal groups or communities?**

◯ Yes ◉ **No**

4.2 Chart Reviews

1.0 **Estimate the number of records you will access and provide the start and end dates of the data pull** *(e.g. We will review approximately 300 charts from December 2005 to November 2009.)*
We will review approximately 243 charts from October 13, 2012 to July 4, 2104.

2.0 **How will you receive the data:**
Data is coded by the study team and a key is maintained separate from the main data.

3.0 **If a member of the study team is pulling the data, does the individual normally have access to the records, eg for clinical purposes?**
(●) **Yes** ( ) No

4.0 **Will individual patient consent be sought or is a waiver of consent required? If requesting a waiver of consent, describe why it is not reasonable, feasible or practical to obtain consent**
A waiver of consent is requested since this is a large group of patients who were seen over a period of 2.5 years. Patients come from across Northern Alberta, BC, Saskatchewan and NWT, and tracking them down for purposes of consent would not be feasible.

5.1 Research Methods and Procedures

Some research methods prompt specific ethic issues. The methods listed below have additional questions associated with them in this application. If your research does not involve any of the methods listed below, ensure that your proposed research is adequately described in Section 2.0: Study Objectives and Design or attach documents in Section 7.0 if necessary.

1.0 * **This study will involve the following** *(select all that apply)*
The list only includes categories that trigger additional page(s) for an online application. For any other methods or procedures, please indicate and describe in your research proposal in the Study Summary, or provide in an attachment:
None of the above

2.0 * **Is this study a Clinical trial? (Any investigation involving participants that evaluates the effects of one or more health-related interventions on health outcomes?**
( ) Yes (●) **No**

3.0 **If you are using any tests in this study diagnostically, indicate the member(s) of the study team who will administer the measures/instruments:**

Test Name	Test Administrator	Organization	Administrator's Qualification
There are no items to display			

4.0 **If any test results could be interpreted diagnostically, how will these be reported back to the participants?**

6.1 Data Collection

1.0 * **Will the researcher or study team be able to identify any of the participants at any stage of the study?**
(●) **Yes** ( ) No

2.0 **Will participants be recruited or their data be collected from Alberta Health Services or Covenant Health or data custodian as defined in the Alberta Health Information Act?**

(•) **Yes** ( ) No

Important: Research involving health information must be reviewed by the Health Research Ethics Board.

3.0 **Primary/raw data collected will be** *(check all that apply)***:**

All personal identifying information removed (anonymized)

4.0 **If this study involves secondary use of data, list all original sources:**

5.0 **In research where total anonymity and confidentiality is sought but cannot be guaranteed** *(eg. where participants talk in a group)* **how will confidentiality be achieved?**

Data will be anonymized prior to analysis and no identifying information will be presented in publications or presentations. All members of the study team have or will receive medical research ethics training that covers the issue of confidentiality.

6.2 Data Identifiers

1.0

* **Personal Identifiers:** will you be collecting - at any time during the study, including recruitment - any of the following *(check all that apply)***:**

Age at time of data collection

If OTHER, please describe:

2.0

Will you be collecting - at any time of the study, including recruitment of participants - any of the following *(check all that apply)***:**

There are no items to display

If OTHER, please describe:

3.0 * **If you are collecting any of the above, provide a comprehensive rationale to explain why it is necessary to collect this information:**

Age is needed as it is an important descriptive variable with some influence on recovery from injury.

4.0 **If identifying information will be removed at some point, when and how will this be done?**

Identifying information will be removed by the Research Director of the GSSMC (Dr. Doug Gross). A master file will be kept on file at the GSSMC,

but the file given used for the analysis will have all identifying information removed.

5.0 * **Specify what identifiable information will be RETAINED once data collection is complete, and explain why retention is necessary. Include the retention of master lists that link participant identifiers with de-identified data:**
A master file with all identifying information will be kept at the GSSMC.

6.0 **If applicable, describe your plans to link the data in this study with data associated with other studies (e.g within a data repository) or with data belongong to another organization:**
None planned.

6.3 Data Confidentiality and Privacy

1.0 * **How will confidentiality of the data be maintained? Describe how the identity of participants will be protected both during and after research.**
As mentioned, data will be anonymized prior to being analyzed by the investigators. We will also avoid presenting identifying information in presentations or publications.

2.0 **How will the principal investigator ensure that all study personnel are aware of their responsibilities concerning participants' privacy and the confidentiality of their information?**
All study team members have been trained on human research ethics including patient privacy and confidentiality. As a large, experienced University of Alberta clinic, the GSSMC has extensive experience with maintaining patient privacy and confidentiality.

3.0

External Data Access

* **3.1 Will identifiable data be transferred or made available to persons or agencies outside the research team?**
○ Yes ◉ **No**

3.2 If YES, describe in detail what identifiable information will be released, to whom, why they need access, and under what conditions? What safeguards will be used to protect the identity of subjects and the privacy of their data.

3.3 Provide details if identifiable data will be leaving the institution, province, or country *(eg. member of research team is located in another institution or country, etc.)*

6.4 Data Storage, Retention, and Disposal

1.0 * **Describe how research data will be stored, e.g. digital files, hard copies, audio recordings, other. Specify the physical location and how it will be secured to protect confidentiality and privacy.** *(For example, study documents must be kept in a locked filing cabinet and computer files are encrypted, etc. Write N/A if not applicable to your research)*
Electronic copies will be provided to the investigators and stored at the GSSMC. All paper files are stored at the GSSMC.

2.0 * **University policy requires that you keep your data for a minimum of 5 years following completion of the study but there is no limit on data retention. Specify any plans for future use of the data. <u>If the data will become part of a data repository or if this study involves the creation of a research database or registry for future research use, please provide details.</u>** <u>*(Write N/A if not applicable to your research)*</u>
We have no future plans, although data will be stored for a minimum of 7 years as the data is contained in patient medical records.

3.0 **If you plan to destroy your data, describe when and how this will be done? Indicate your plans for the destruction of the identifiers at the earliest opportunity consistent with the conduct of the research and/or clinical needs:**
No plans to destroy the data as it is part of the patients' medical records.

7.1 Documentation

Add documents in this section according to the headers. Use Item 11.0 "Other Documents" for any material not specifically mentioned below.

Sample templates are available in the REMO Home Page in the **Forms and Templates**, or by clicking HERE.

1.0 **Recruitment Materials:**

Document Name	Version	Date	Description
There are no items to display			

2.0 **Letter of Initial Contact:**

Document Name	Version	Date	Description
There are no items to display			

3.0 **Informed Consent / Information Document(s):**

3.1 What is the reading level of the Informed Consent Form(s):

3.2 Informed Consent Form(s)/Information Document(s):

Document Name	Version	Date	Description
There are no items to display			

4.0 Assent Forms:

Document Name	Version	Date	Description
There are no items to display			

5.0 Questionnaires, Cover Letters, Surveys, Tests, Interview Scripts, etc.:

Document Name	Version	Date	Description
There are no items to display			

6.0 Protocol:

Document Name	Version	Date	Description
There are no items to display			

7.0 Investigator Brochures/Product Monographs *(Clinical Applications only)***:**

Document Name	Version	Date	Description
There are no items to display			

8.0 Health Canada No Objection Letter *(NOL)***:**

Document Name	Version	Date	Description
There are no items to display			

9.0 Confidentiality Agreement:

Document Name	Version	Date	Description
There are no items to display			

10.0 Conflict of Interest:

Document Name	Version	Date	Description
There are no items to display			

11.0 Other Documents:

For example, Study Budget, Course Outline, or other documents not mentioned above

Document Name	Version	Date	Description
There are no items to display			

Final Page

You have completed your ethics application! Please select "Exit" to go to your study workspace.

This action will NOT SUBMIT the application for review.

Only the Study Investigator can submit an application to the REB by selecting the "SUBMIT STUDY" button in My Activities for this Study ID:

Pro00049954 .

You may track the ongoing status of this application via the study workspace.

Please contact the REB Coordinator with any questions or concerns.
